# Evaluation of suitable reference genes for gene expression studies in bronchoalveolar lavage cells from horses with inflammatory airway disease

**DOI:** 10.1186/1471-2199-12-5

**Published:** 2011-01-28

**Authors:** Laura Beekman, Triin Tohver, Rkia Dardari, Renaud Léguillette

**Affiliations:** 1 Departement of Veterinary Clinical and Diagnostic Sciences, Faculty of Veterinary Medicine, University of Calgary, 3330 Hospital Drive NW, Calgary, Alberta, T2N 4N1, Canada; 2 Departement of Microbiology and Infectious Diseases, Faculty of Medicine, University of Calgary, 3330 Hospital Drive NW, Calgary, Alberta, T2N 4N1, Canada

## Abstract

**Background:**

The stability of reference genes has a tremendous effect on the results of relative quantification of genes expression by quantitative polymerase chain reaction. Equine Inflammatory Airway Disease (IAD) is a common condition often treated with corticosteroids. The diagnosis of IAD is based on clinical signs and bronchoalveolar lavage (BAL) fluid cytology. The aim of this study was to identify reference genes with the most stable mRNA expression in the BAL cells of horses with IAD irrespective of corticosteroids treatment.

**Results:**

The expression stability of seven candidate reference genes (B2M, HPRT, GAPDH, ACTB, UBB, RPL32, SDHA) was determined by qRT-PCR in BAL samples taken pre- and post- treatment with dexamethasone and fluticasone propionate for two weeks in 7 horses with IAD. Primers' efficiencies were calculated using LinRegPCR. NormFinder, GeNorm and qBasePlus softwares were used to rank the genes according to their stability. GeNorm was also used to determine both the ideal number and the best combination of reference genes. GAPDH was found to be the most stably expressed gene with the three softwares. GeNorm ranked B2M as the least stable gene. Based on the pair-wise variation cut-off value determined with GeNorm, the number of genes required for optimal normalization was four and included GAPDH, SDHA, HPRT and RPL32.

**Conclusion:**

The geometric mean of GAPDH, HPRT, SDHA and RPL32 is recommended for accurate normalization of quantitative PCR data in BAL cells of horses with IAD treated with corticosteroids. If only one reference gene can be used, then GAPDH is recommended.

## Background

Inflammatory Airway Disease (IAD) is a non-septic lung disease defined for the first time in 2002 [1] and that affects the lower airways in horses [2]. The syndrome is later defined in a consensus publication [2] as moderate lower airway neutrophilic inflammation or any lower airway inflammation with mast or eosinophilic cells, not associated with signs of labored breathing at rest. IAD affects a large number of horses and can impede their performance [3-5]. Because the IAD phenotype has only been described recently, its pathophysiology is still not understood and is under investigation. Knowledge of molecular mechanisms underlying this disease is a fundamental prerequisite to understand the etiology and the underlying inflammatory mechanism involved in IAD. Recently, the first evidence for a corticosteroids treatment suppressing the airway hyperreactivity featured in IAD has been established (Tohver T., New D., Nicol J., McDonald K., Fernandez N., Léguillette R.: Dexamethasone and fluticasone significantly decrease airway hyperresponsiveness in horse with inflammatory airway disease (IAD), submitted). Tohver et al. found a significant reduction in airway hyperreactivity and airway hypersensitivity after treatment with either intramuscular dexamethasone or inhaled fluticasone propionate in horses with IAD. However, neither of the treatments affected the differential cell count in the bronchoalveolar lavage fluid (BALF). Understanding the pathophysiology of IAD as well as the mechanism of action of corticosteroids in this disease implies a better understanding of the inflammatory cells activity. Two studies have reported the expression of inflammatory cytokines and chemokines in inflammatory cells from the BALF in horses with recurrent airway obstruction after treatment with corticosteroids [6,7], but this is still to be studied in IAD.

Real-time quantitative PCR is a standard method for accurate, sensitive and rapid quantification of gene expression nowadays [8,9]. Relative quantification using PCR allows comparing genes expression between groups, for example before and after a treatment. When analyzing data for relative quantification, results are normalized to a reference. Normalization is extremely important to allow accurate comparison of the results between different samples and conditions in gene expression studies [10]. There have been a lot of different strategies proposed for normalizing, that range from ensuring that a similar sample size is chosen to the use of an internal reference gene [10]. Normalizing to a reference gene is a widely used method because it is simple in theory. An ideal reference gene should be stably expressed and unaffected by experimental protocol or disease status [11]. Commonly used reference genes such as GAPDH and β-actin are unfortunately often used without prior validation of their expression stability under the specific study conditions. However, a number of studies have shown that the expression of those genes is significantly altered in some experimental conditions [12-15]. It is therefore necessary to validate the expression stability of reference genes prior to their use in an experimental protocol. Ideally, it has been recently recommended that a combination of reference genes should be used to obtain a more stable reference [16].

Regarding horses, a number of potential reference genes have been studied in different tissues and cell cultures including normal skin and sarcoids [17], colon, heart, kidney, liver, lung, lymph node, small intestine and spleen [18] and also in peripheral blood mononuclear cells [18-20]. However, although many studies are using the BALF as a sample of choice to study the activity of cells in the lungs, the most reliable reference genes in equine BALF have not been studied. In addition, robust reference genes are needed when using corticosteroids because of the extensive effects these medications have on cellular metabolism.

The aim of this study was therefore to validate reference genes for gene expression studies in BALF of horses with IAD irrespective of treatment with dexamethasone (DEX) or fluticasone propionate (FLUC).

## Results

### Bronchoalveolar lavage fluid total and differential cell counts

**Figure 1:**
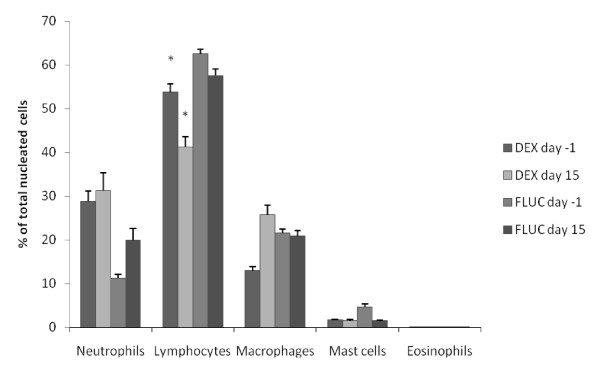
**Bronchoalveolar lavage fluid differential cell counts in horses with IAD pre- and post- treatment with corticosteroids**. DEX indicates dexamethasone. FLUC indicates fluticasone. Bars indicate standard deviation. The figure shows the differential cell counts for neutrophils, lymphocytes, macrophages, mast cells and eosinophils as a percentage of the total amount of nucleated cells. * indicates a significant difference between pre- and post- treatment (p < 0.05).

The results of the BALF cytological analysis are shown in figure 1 and table 1. The BALF total and differential cell counts were not significantly different between the DEX and FLUC treatment baseline values. The percentage lymphocytes in the BALF decreased significantly after DEX (p = 0.039) (figure 1 and table 1). Treatment with DEX and FLUC did not induce any significant change in total cell count or differential cell count for any other cell type in the BALF.

**Table 1:** BALF cell counts in 7 horses with IAD before and after treatment with corticosteroids.

	Lymphocytes (%)	Macrophages (%)	Neutrophils (%)	Mast cells (%)	Eosinophils (%)	Total Cell Count (cells/mm^3^)
**Dexamethasone**						
**Day 1**						
**Horse 1**	56.17	29.14	13.84	0.36	0.18	298.9
**Horse 2**	53.35	27.02	17.78	1.85	0.00	441.1
**Horse 3**	30.09	62.39	5.69	1.65	0.18	150.0
**Horse 4**	45.49	30.45	3.95	3.95	0.00	113.3
**Horse 5**	68.09	9.98	21.03	0.98	0.00	231.1
**Horse 6**	56.83	21.39	17.82	1.98	0.00	142.2
**Horse 7**	66.52	21.23	10.94	1.31	0.00	122.2

**Mean ± SEM**	53.79 ± 4.91 *	28.80 ± 6.17	13.01 ± 2.44	1.73 ± 0.43	0.05 ± 0.01	214.1 ± 45.5
**Day 15**						
**Horse 1**	52.74	16.44	29.45	1.37	0.00	212.2
**Horse 2**	17.01	78.07	4.30	0.20	0.20	1026.7
**Horse 3**	22.12	62.82	15.06	0.00	0.00	204.4
**Horse 4**	40.72	13.80	40.50	4.75	0.00	90
**Horse 5**	48.16	1.64	46.72	3.48	0.00	162.2
**Horse 6**	52.21	15.50	32.10	0.18	0.00	35.6
**Horse 7**	56.30	30.87	12.17	0.65	0.00	110

**Mean ± SEM**	41.32 ± 5.94 *	31.31 ± 10.73	25.76 ± 5.92	1.52 ± 0.71	0.03 ± 0.01	263.0 ± 129.5

**Fluticasone Day 1**						
**Horse 1**	66.42	13.18	20.40	0.00	0.00	246.7
**Horse 2**	75.55	7.64	14.30	2.51	0.00	378.9
**Horse 3**	60.68	11.14	17.50	10.45	0.23	42.2
**Horse 4**	54.39	6.34	35.12	4.15	0.00	104.4
**Horse 5**	54.84	14.75	19.12	11.29	0.00	167.8
**Horse 6**	67.00	5.30	24.60	3.30	0.00	48.9
**Horse 7**	58.50	20.70	19.60	1.10	0.00	93.3

**Mean ± SEM**	62.48 ± 2.89	11.29 ± 2.06	21.52 ± 2.55	4.69 ± 1.68	0.03 ± 0.01	154.6 ± 46.0
**Day 15**						
**Horse 1**	69.34	8.42	21.84	0.40	0.00	156.7
**Horse 2**	45.35	45.35	7.88	1.41	0.00	143.3
**Horse 3**	62.71	14.29	22.76	0.24	0.00	123.3
**Horse 4**	58.33	14.47	23.90	3.07	0.22	133.3
**Horse 5**	40.04	47.00	10.83	2.13	0.00	n/a
**Horse 6**	60.60	1.10	36.00	1.90	0.40	20.1
**Horse 7**	66.40	9.10	23.00	1.50	0.00	56.7

**Mean ± SEM**	57.54 ± 4.11	19.96 ± 6.98	20.89 ± 3.51	1.52 ± 0.37	0.09 ± 0.02	105.6 ± 22.2

* indicates a significant difference between pre- and post- treatment (p < 0.05).

### Purity and quantity of extracted RNA

The optical density (OD) ratio A_260_/A_280 _nm measured with a Nanodrop spectrophotometer was 1.95 ± 0.12 (OD A_260_/A_280 _ratio ± SD). The average RNA concentration after extraction using the RNeasy Mini Kit was 57.14 μg/μl ± 41.48 (μg/μl ± SD).

### Amplification efficiency

The amplification efficiencies for individual reactions were calculated using the LinRegPCR software. The results of the averaged efficiencies are shown in table 2. The amplification efficiencies for the seven candidate reference genes ranged between 96.15% ± 5.74 (efficiency % ± SD) and 103.45% ± 5.54 (efficiency % ± SD).

**Table 2:** Primers information and PCR reactions efficiencies for candidate reference genes.

Gene	Accession Number	Oligo	Sequence	Amplicon size (bp)	Efficiency ± SD (%)
ACTB	AF035774	Forward	CTGGCACCACACCTTCTACA	249	96.15 ± 5.74
		Reverse	CCCTCATAGATGGGCACAGT		
GAPDH	AF083897	Forward	GGTGAAGGTCGGAGTAAACG	106	96.25 ± 4.63
		Reverse	AATGAAGGGGTCATTGATGG		
B2M	X69083	Forward	CCTGCTCGGGCTACTCTC	89	103.45 ± 5.54
		Reverse	CATTCTCTGCTGGGTGACG		
HPRT	AY372182	Forward	AATTATGGACAGGACTGAACGG	121	101.05 ± 3.27
		Reverse	ATAATCCAGCAGGTCAGCAAAG		
RPL32	CX594263	Forward	GGGAGCAATAAGAAAACGAAGC	138	101.25 ± 2.45
		Reverse	CTTGGAGGAGACATTGTGAGC		
SDHA	DQ402987	Forward	GAGGAATGGTCTGGAATACTG	91	100.5 ± 1.96
		Reverse	GCCTCTGCTCCATAAATCG		
UBB	AF506969	Forward	TTCGTGAAGACCCTGACC	91	100.65 ± 2.35
		Reverse	CCTTATCCTGGATCTTGGC		

Primers sequences were determined using primer3 software and Ensembl Genome Browser (EMBL accession numbers are indicated). Efficiencies are averages of all individual efficiencies calculated for each reaction using LinReqPCR software.

### Expression levels of candidate reference genes

**Figure 2:**
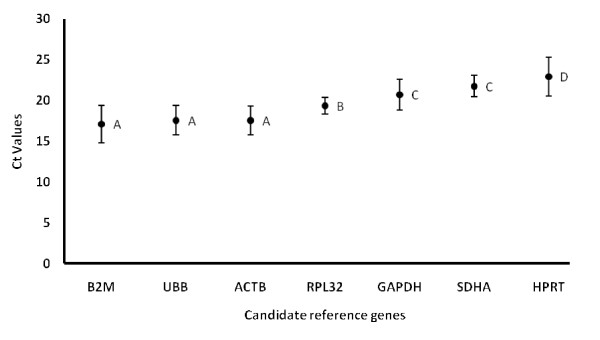
**Average cycle threshold (Ct) values of candidate reference genes tested in the bronchoalveolar lavage fluid of horses with IAD**. The values are RT-PCR cycle threshold numbers (Ct values) pre-and post-treatment in horses with IAD treated with corticosteroids. The bars indicate standard deviation. Letters indicate a significant difference in average Ct value. Average Ct values that have the same letter are not significantly different.

The cycle threshold (Ct) values obtained throughout the study were low enough to pursue the analysis reliably: Overall and by combining pre- and post-treatment Ct values for each gene, out of the seven genes studied, B2M (mean Ct 17.08) and UBB (mean Ct 17.56) were expressed at the highest levels, followed by ACTB (mean Ct 17.56), RPL32 (mean Ct 19.36), GAPDH (mean Ct 20.72) and SDHA (mean Ct 21.74). HPRT (mean Ct 22.95) was expressed at the lowest level in the cells of the BALF (figure 2). Prior to any referencing, there was no significant difference in average Ct value between SDHA and GAPDH as well as between β-actin, UBB and B2M. Also, the average Ct values for HPRT and RPL32 were significantly different from all the other candidate reference genes.

### Identification of optimal reference genes

**Figure 3:**
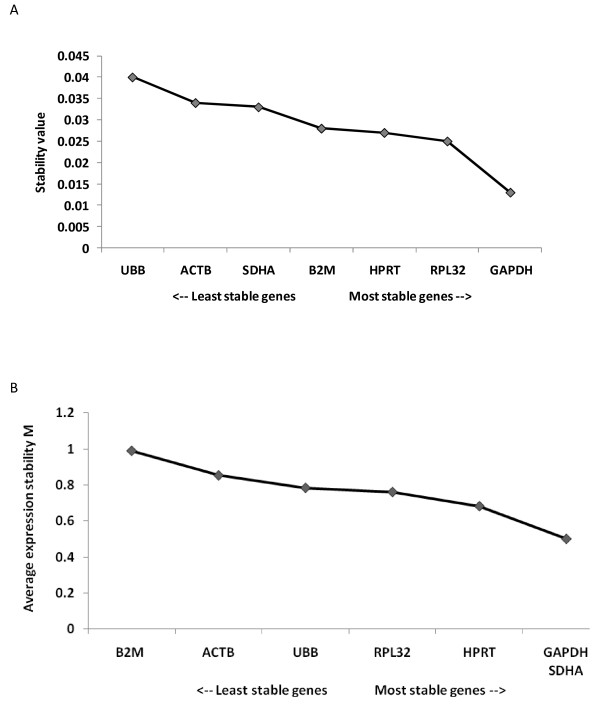
**Ranking of seven candidate reference genes using NormFinder and GeNorm softwares**. (A) NormFinder ranks the genes based on a calculated stability value. The lower the stability value, the higher the expression stability. (B) GeNorm ranks the candidate reference genes based on their stability parameter M. The lower the M value, the higher the expression stability.

Figure 3A shows the ranking of the seven candidate reference genes based on their stability values calculated using NormFinder. GAPDH has the lowest stability value and therefore is the most stably expressed gene (stability value: 0.013) followed by RPL32 (stability value: 0.025), HPRT (stability value: 0.027), B2M (stability value: 0.028), SDHA (stability value: 0.033) and ACTB (stability value: 0.034). UBB has the highest stability value (at 0.04) and is therefore the least stably expressed. The best combination of two genes was GAPDH and RPL32 with a stability value of 0.014.

Figure 3B shows the ranking of the seven candidate reference genes based on their M value calculated using GeNorm. All the candidate reference genes started with an M value below 1.5, which is the default limit below which candidate reference genes can be classified as stably expressed. GeNorm ranks GAPDH and SDHA as the most stably expressed candidate reference genes, followed by HPRT, RPL32 and UBB. B2M and ACTB were the least stably expressed candidate reference genes.

The qBaseplus software was used to confirm the results obtained with GeNorm and NormFinder softwares. The unstable genes B2M and ACTB were not included in the evaluation. Based on the coefficient of variation (CV) value calculated in qBaseplus GAPDH was, in agreement with GeNorm and NormFinder, the most stably expressed gene in the study (table 3).

**Table 3:** Candidate reference genes stability results using qBaseplus software.

Reference target	M value	CV value
GAPDH	0.695	0.274
HPRT	0.830	0.476
RPL32	0.781	0.364
SDHA	0.828	0.388
UBB	0.816	0.347

M values, which is a parameter for the gene stability, and Coefficient of Variation (CV) values, which indicates how stably a gene is expressed, show that GAPDH is the most stably expressed gene.

### Determination of the optimal number of reference genes for normalization

**Figure 4:**
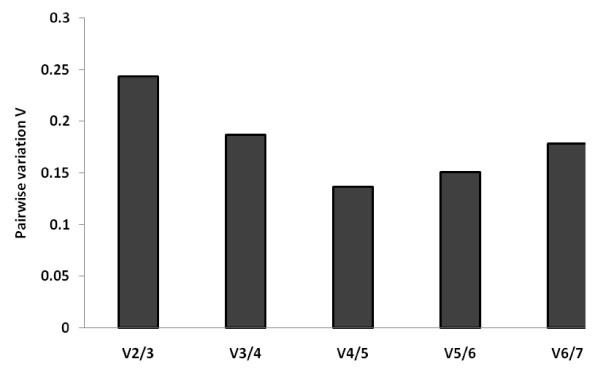
**Determination of the optimal number of reference genes for normalization**. The GeNorm software calculates the normalization factor from an increasing number of genes (starting with at least two) for which the variable V defines the pairwise variation between two sequential normalization factors. The lower the pairwise variation, the better is the combination of genes for reference. V4/5 for example, shows the variation between the normalization factors of four genes in relation to five genes and shows that four genes is the combination providing the lowest pairwise variation.

In addition to the stability results, the GeNorm software can determine the optimal number of reference genes necessary to calculate a normalization factor. The results are shown in figure 4. Only a combination of the four most stable genes GAPDH, SDHA, HPRT and RPL32, have a pairwise variation value lower than the cut off value of 0.15. In addition, there is a very good agreement between GeNorm and NormFinder softwares identifying three out of four most stable genes, namely GAPDH, HPRT and RPL32. We therefore conclude that the combination of GAPDH, HPRT, SDHA and RPL32 is the most appropriate normalization approach for gene expression studies in BALF of horses with inflammatory lung diseases treated with corticosteroids.

## Discussion

Using reference genes that have a stable expression between the compared groups is crucial in gene expression studies. Several studies have shown that the use of different reference genes can change the outcome and conclusions of a study [21-25]. The aim of the present study was therefore to validate, for the first time, reference genes for studies in BALF of horses with IAD, irrespective of treatment with corticosteroids. We found that using GAPDH, HPRT, SDHA and RPL32 as a combination of reference genes is the most appropriate normalization approach in this experimental design and that GAPDH is the single most stably expressed gene in the BALF of horses treated with corticosteroids. Although the present study only used 7 horses, which is less than typical studies using mice or human samples, it is comparable to other clinical studies in equine medicine using PCR on 3 to 10 horses [17,19,20,26,27]. Compared to other techniques measuring gene expression, the PCR technique is better suited for samples of smaller size and the number of horses used here allowed obtaining significant findings that are relevant for studies in the field of equine medicine.

To our knowledge, there are only two studies evaluating the stability of reference genes in horse tissues, one being in the skin [17] and the other being in the peripheral blood [19]; no data is available on the stability of reference genes in the lungs of horses to compare our results with. However, Cappelli at al. found GAPDH as the least stably expressed gene in the panel of candidate reference genes they tested in equine blood lymphocytes during exercise-induced stress [19]. This emphasizes the importance of appropriate reference gene validation for every tissue and experimental protocol, even when using the same species. The discrepancy is probably due in this case to the difference in tissues tested as well as to the effect of corticosteroids on cellular metabolism. In addition, a few studies assessed the validity of proposed housekeeping genes in the bronchoalveolar cells of humans with various pathologies [21,28,29]. Using a different method than reported here, a study found that GAPDH was the most stable reference gene in the bronchoalveolar samples of people with nonsmall cell lung cancer [29]. Another study used Genorm to test candidate housekeeping genes that were mostly different from those described here and found that GNB2L1, HPRT1 and RPL32 were the most stably expressed genes in alveolar macrophages from 22 subjects with chronic obstructive pulmonary disease (COPD) [28]; they also described that GAPDH was inappropriate for these studies. In agreement, one study found that GAPDH and ACTB were not suitable as reference genes in asthma and illustrated it by showing that the use of GAPDH vs ACTB as reference genes would lead to conflicting results [22]. Lastly, a study used equivalence test as well as the statistical tools BestKeeper, GeNorm and NormFinder to assess the most suitable housekeeping genes in the lungs of a large number of people (2 cohort studies) irrespective of gender, smoking, lung pathologies, treatments, and BAL cytology [21]. This study only shared 3 reference genes with the data presented here, but found that only RPL32 along with the proteasome subunit 2 (PSMB2) were stably expressed among BAL samples [21]. In this sense, RPL32 came as the second and third most stably expressed gene in the NormFinder and GeNorm analysis presented here.

Numerous methods are available to validate reference genes for relative quantification by QPCR. Studies will often use only one method, but one study compared two reference genes validation methods in horse samples [19] and another compared four methods in humans BAL samples [21]. Similarly, to ensure consistency and for comparison purpose, the data was analyzed here using three different softwares (GeNorm, NormFinder and qBasePlus). GeNorm and qBasePlus use a pairwise comparison model, while NormFinder uses a model-based approach. In our study, GeNorm 1) identified GAPDH, SDHA, HPRT and RPL32 as the most stably expressed reference genes by calculating a stability parameter (M) (see methods) and 2) determined that the optimal number of reference genes to be used was 4 (GAPDH, SDHA, HPRT and RPL32), by calculating pair-wise variation (V values) (see methods). NormFinder defined GAPDH as the best reference gene when using the treatment with corticosteroids as group identifiers to calculate a stability value for each candidate reference gene. NormFinder takes into account variation across subgroups, thus avoiding artificial selection of co-regulated genes by analyzing the expression stability of candidate genes independently from each other [16]. Lastly, qBasePlus confirmed that GAPDH is the best reference gene in our study design. Similarly to previous studies [19,30], we found a good agreement in the reference genes ranking between GeNorm and NormFinder as they both ranked GAPDH as the most stable expressed gene, which was confirmed by the qBasePlus analysis. We also found that the first three most stable reference genes were consistently the same when using GeNorm and NormFinder, even if they were not in the exact same ranking order. There was a slight difference in the top four most stably expressed genes as the four most stably expressed genes ranked by NormFinder were, in decreasing order, GAPDH, RPL32, HPRT and B2M, while they were GAPDH, SDHA, HPRT and RPL32 with GeNorm. Very similar discrepancy between the different algorithms has been observed in other studies comparing statistical analysis methods: The only study using horse samples and comparing GeNorm with NormFinder found also that the best three reference genes were ranked differently by the two algorithms and that there was disagreement on the fourth most stable gene [19]. Another study using BAL samples on a large cohort of human patients also found different ranking order and genes identification for the top four most stable reference genes [21]. Such discrepancy could be explained by genes' co-regulation. Indeed, co-regulated genes may become highly ranked independently of their expression stabilities with GeNorm software [31]. In contrast, results obtained with NormFinder are not significantly affected by co-regulation of candidate reference genes. In our study, the main difference in ranking involved SDHA, which ranked as the fifth most stable gene with NormFinder, but ranked second with GeNorm. Although co-regulation has been described for ACTB, B2M, GAPDH and HPRT, we did not find evidence for the possible co-regulation of SDHA in the literature. To check for possible co-regulation of SDHA, we analyzed the data again with GeNorm, this time excluding SDHA from the candidate reference gene panel. We found that removing SDHA from the analysis did not resolve the discrepancy in ranking of the candidate reference genes between NormFinder and GeNorm (data not shown). We thus concluded that the discrepancy in ranking was not caused by co-regulation of SDHA.

ACTB and UBB were ranked by both softwares as unstably expressed genes and therefore should not be used as reference in gene expression studies in bronchoalveolar lavage (BAL) cells obtained from horses with IAD, which is similar (for ACTB) as another study using humans BAL samples [21]. It is however in contrast with a study done in normal horses' skin in which ACTB and UBB came out as the most stably expressed genes from the panel tested with GeNorm [17]. GAPDH was however not evaluated in this previous study [17]. This shows again that the choice of reference genes cannot be transposed from on study to the other without validation for the specifics of each experimental protocol.

As described above, GeNorm also provides a measure for the best number of reference genes that should be used for optimal normalization. In agreement with several previous studies, we found that the use of more than one reference gene allows for a more accurate normalization than the use of only one reference gene [10,16,31]. Based on a cut-off point of 0.15 for the V value, as described by Vandesompele et al [16], a combination of the four most stable reference genes was calculated as being optimal for gene expression studies in BAL cells of horses with IAD treated with corticosteroids (figure 4).

## Conclusion

In conclusion, we recommend using the geometric mean of GAPDH, HPRT, SDHA and RPL32 to guarantee suitable normalization in bronchoalveolar lavage cells obtained from horses with IAD treated with either DEX or FLUC. If only one reference gene can be used, GAPDH should be chosen because it is unanimously the most stably expressed reference gene tested for this type of studies.

## Methods

This study was approved by the Animal Care Committee of the Health Science Centre at the University of Calgary. The authors used the REFLECT statement guidelines to report this study [32].

### Sample collection

Bronchoalveolar lavage (BAL) samples were taken from 7 horses with IAD. The number of horses was calculated using a power of 0.9 for a difference in measured parameters between baseline and treatments of 2 times the within-patient standard deviation. Horses were mixed breeds, 4 mares, 3 geldings of various ages (range: 4-16 year old). Criteria for inclusion followed the recommendations from a consensus publication and were: 1- the presence of respiratory clinical signs during exercise without labored breathing at rest 2- the absence of increased lung resistance at rest after a challenge with moldy hay 3- the presence of airway hyperreactivity measured by an increase in lung resistance (R_L_) by 75% at lower doses of nebulized histamine 4- a BAL with increased percentage of mast cells (> 2%) and/or eosinophils (>0.1%) and/or neutrophils (>10%). The study used a randomized cross-over design as follow: The 7 horses were randomly divided into two groups (using Microsoft^® ^excel randomization function) which were each subjected to two treatment protocols sequentially but separated in the middle by a three weeks washout period. A baseline BAL was performed as described below before each treatment period on day -1. Dexamethasone (0.05 mg/kg) was administered intra muscularly once a day in the morning (between 7:00 and 8:00 AM) for 15 days. Fluticasone (3000 μg) was administered by inhalation using an Aerohippus^® ^twice daily (between 7:00 and 8:00 AM and PM) for 15 days. A second BAL was performed on day 15.

The BAL procedure was done as previously described [5]. Briefly, the BAL was performed after sedating the horses using a flexible videoendoscope that was passed through a nostril and directed into the lung until it was wedged into the main bronchus. An instillation of lidocaine solution through the channel of the endoscope was done to desensitize the airway mucosa during the progression of the endoscope. After wedging of the endoscope 250 ml of 0.9% sterile sodium chloride was instilled into the bronchus and aspirated via the endoscope biopsy channel by use of a suction pump and collected in a 500 ml plastic Nalgene^® ^jar. This process was repeated with a second bolus of sterile saline. Samples were immediately stored at 4°C until further processing in the laboratory which was done within 2 hours of the BAL procedure.

Slides were prepared for the total and differential cell counts using a sample of BAL fluid that had been put in a vacutainer^® ^EDTA tube right after the BAL procedure. Slides were prepared using a cytospin^® ^(1000 rpm for 4 minutes) and stained using an automatic stainer (Hema-Tek^® ^2000, Bayer) with a Modified Wright Giemsa solution for better visualization of the mast cells. Differential counts were performed on at least 400 nucleated cells, not including epithelial cells.

Two 50 ml aliquots of BALF were centrifuged at 700 g for 10 minutes (GP Centrifuge, Beckman, USA) before removing the supernatant and resuspending the cells pellet in 1.5 ml of RNAlater (Qiagen, Mississauga, Ontario, Canada). The samples were immediately frozen at -80°C.

### RNA extraction

The RNA extractions were performed at a later time (approximately 3 months after the BAL procedure). The samples were thawed at room temperature, centrifugated, and the RNAlater supernatant was aspirated. The cells from the pellet were homogenized using the needle and syringe method. Total RNA was extracted using the RNeasy mini kit (Qiagen) according to the manufacturer's recommendations. The yield and the purity of the extracted RNA were measured using the Nanodrop ND-1000 spectrophotometer (Thermoscientific, Wilmington, USA) by optical density (OD) A_260_/A_280 _nm.

### First-strand cDNA synthesis

An average of 435 ± 109.6 ng (± SD) total RNA for each sample was retro-transcribed immediately after the RNA extraction using the Omniscript RT Kit (Qiagen) combined with Oligo(dT)_12-18 _Primers (Invitrogen, Burlington, Ontario, Canada) and RNaseOUT Recombinant Ribonuclease Inhibitor (Invitrogen) according to the manufacturer's specifications. cDNA was stored at -80°C until further use.

### Reference gene selection and primer design

No previous study validated reference genes in the BAL fluid of horses, and more "traditional" reference genes like GAPDH and ACTB have been most often used in equine respiratory medicine [33-38]. Regarding other equine organs, HPRT, B2M, SDHA, UBB, and RPL32 have been previously compared in peripheral blood [19], and rNA 18S, 28S, B2M, ACTB, GAPDH and HPRT1 have been studied by one group on various horse tissues [18]. The genes selected in our study were based on these previous studies and were from different functional classes to minimize the possibility of co-regulation. Designing primers for reference genes is more challenging in horses than in laboratory animals or humans because of the less detailed sequences information available. Information about the candidate reference genes used in the present study is shown in table 2. The following seven commonly used reference genes were selected: β-actin (ACTB), glyceraldehyde-3P-dehydrogenase (GAPDH), hypoxanthine ribosyltransferase (HPRT), β-2-microglobin (B2M), succinate dehydrogenase complex subunit A (SDHA), ubiquitin B (UBB) and ribosomal protein L32 (RPL32). Primers for equine HPRT, B2M, SDHA, UBB, and RPL32 were previously described [19]. Primers for ACTB and GAPDH were designed using the Primer3 software [39] based on horse sequences from the Ensembl Genome Browser [40]. The primers were designed so that the predicted amplicons would span exon-exon boundaries. They were tested using a BLAST analysis against the Ensembl database and verified using MFold [41]. The optimal primer annealing temperatures were determined on cDNA from BAL samples obtained from horses with IAD. Melting curve analysis as well as agarose gel electrophoresis were performed to test for the specificity of the amplicons.

### Real-time quantitative PCR

One microliter of cDNA was added to 13 μl PerfeCta™ SYBR^® ^Green Super Mix Low ROX, 40 nM of the forward primer, 40 nM of the reverse primer and 7 μl of nuclease free water to a final volume of 25 μl. The PCR reactions were performed on a MX3005P machine (Stratagene, La Jolla, CA, USA). PCR conditions were: Initial denaturation at 95°C for 5 minutes to activate the DNA polymerase, followed by 45 cycles of denaturation at 95°C for 1 minute, annealing at the primer specific annealing temperature for 30 seconds and extension at 70°C for 30 seconds. After the last cycle the melting curve was determined in the range 60°-95°C. For GAPDH and ACTB we used the same protocol, only the annealing temperature was 64°C instead of 62°C. All the reactions were executed in triplicate. Negative control samples were always included in each run to check for contamination.

### Data analysis

The raw RT-QPCR amplification data was exported from the MxPro^® ^software (Stratagene) to Microsoft^® ^excel. The software LinRegPCR was used to calculate the efficiencies for all the reactions individually. LinRegPCR is a freeware that uses non-baseline corrected data to perform a baseline correction on each sample, then determines a window-of-linearity and finally uses linear regression analysis to fit a straight line trough the PCR data set. From the slope of this line the PCR efficiency of each individual sample is calculated [42]. The average of the individual efficiencies for each candidate reference gene was used for the gene expression levels analysis. The efficiency-corrected Ct-values were converted to a linear scale using the ΔCt-method. The averages of the ΔCt-values for each triplicate were used for stability comparison of candidate reference genes in the NormFinder, GeNorm and qBasePlus softwares.

NormFinder uses an ANOVA-based model [31]. The software calculates a stability value for all candidate reference genes tested. The stability value is based on the combined estimate of intra- and inter-group expression variations of the genes studied. A low stability value, indicating a low combined intra- and inter-group variation, indicates high expression stability.

GeNorm calculates the stability using a pairwise comparison model [16]. This program selects the two most stable genes or a combination of multiple stable genes from a panel of candidate reference genes for normalization. The ranking of the genes is based on the gene stability parameter M. Stepwise exclusion of the gene with the highest M value and recalculation results in a ranking of the candidate reference genes. Lower M values represent higher expression stabilities. To determine the optimal number of reference genes required for accurate normalization, the program calculates the pairwise variation V_n/n+1 _between sequential normalization factors containing an increasing number of reference genes. A large variation means that the added gene has a significant effect and should preferably be included for calculation of a reliable normalization factor. If V_n/n+1 _< 0.15 the inclusion of an additional reference gene is not required and the recommended number of reference genes is given by n.

qBasePlus is a program designed for qPCR data management and analysis and was developed based on algorithms from GeNorm and qBase [43]. To determine the expression stability of the candidate reference genes qBasePlus calculates an M value, similar to the one calculated by the GeNorm software, for all the candidate genes. In addition, the qBasePlus software calculates a coefficient of variation (CV) for all the genes. This CV value indicates how stably each gene is expressed. We used this program to confirm and compare it with the results obtained by the GeNorm and Normfinder software. We used the cut-off values of 0.7 and 40% respectively for the M and CV values below which a gene is considered stably expressed. Only the expression stability of the 5 most stable expressed candidate reference genes (GAPDH, HPRT, RPL32, SDHA and UBB) was evaluated using this program.

### Statistical analysis

Statistical analysis of reference genes stability and ranking was provided by GeNorm, NormFinder, and qBasePlus. In addition, differences in average Ct values of candidate reference genes were calculated using an ANOVA followed by a Bonferroni Pairwise Comparison Test. Differences in BAL differential cell counts before/after treatment were calculated using a Wicoxon Signed Rank test. P values of less than 0.05 were considered significant.

## Abbreviations

BAL: bronchoalveolar lavage; BALF: bronchoalveolar lavage fluid; M: GeNorm gene stability parameter; CV: coefficient of variation; ACTB: β-actin; GAPDH: glyceraldehyde-3p-dehydrogenase; HPRT: hypoxanthine ribosyltransferase; B2M: β-2-microglobulin; SDHA: succinate dehydrogenase complex subunit A; UBB: ubiquitin B; RPL32: ribosomal protein L32; DEX: dexamethasone; FLUC: fluticasone propionate; IAD: inflammatory airway disease; Ct: cycle threshold;

## Authors' contributions

All authors read and approved the final manuscript. LB designed the primers, performed the QPCR experiments, gels and analysis. TT performed the BALs and treatments. RD contributed to the QPCR troubleshooting and experiments. RL conceived, designed and coordinated the study.
